# Role of CD146 Enrichment in Purification of Stem Cells Derived from Dental Pulp Polyp 

**DOI:** 10.22037/iej.2017.19

**Published:** 2017

**Authors:** Maryam Sadat Tavangar, Seyed-Mojtaba Hosseini, Ali Dehghani-Nazhvani, Ahmad Monabati

**Affiliations:** a *Department of Operative Dentistry, Dental School, Shiraz University of Medical Sciences, Shiraz, Iran; *; b * Student Research Committee,Cellular and Molecular Research Club, Shiraz University of Medical Sciences, Shiraz, Iran; *; c * Cardiovascular Research Center, Shiraz University of Medical Sciences, Shiraz, Iran; *; d * Department of Oral Pathology, Dental School, Shiraz University of Medical Sciences, Shiraz, Iran; *; e *Hematology Research Center, Shiraz University of Medical Sciences, Shiraz, Iran; *; f *Molecular Pathology Research Center, Shiraz University of Medical Sciences, Shiraz, Iran*

**Keywords:** Adult Stem Cell, CD146, Dental Pulp, Dental Pulp Disease, Pulpitis, Pulp Polyp, Stem cell Assay

## Abstract

**Introduction:**

Hyperplastic pulpitis (pulp polyp) tissues contains cells with stem cell properties similar to that of the dental pulp stem cells (DPSCs). It has also been shown that CD146 enrichment can homogenize the cultures of DPSCs and enhance the colony forming potentials of their cultures. This study determines whether CD146 enrichment can help purifying the stem cells from heterogeneous cultures of the pulp polyp derived stem cells (PPSCs).

**Methods and Materials:**

Healthy dental pulps and pulp polyp tissues were enzymatically digested and the harvested single cells were sorted according to the presence of CD146 marker. The sorted cells were seeded directly for colony forming unit (CFU) assays of the negative and positive portions. Flowcytometric antigen panel and differentiation assays were used to see if these cells conform with mesenchymal stems cells (MSCs) definition. Differences between the between groups was assessed using independent t-test. The level of significance was set at 0.05.

**Results:**

Normal pulp tissue derived cells formed higher colonies (42.5±16.8 per 10^4 ^cells) than the pulp polyp (17.75±8.9 per 10^4^ cells) (*P*=0.015). The CD146 positive portion of the polyp derived cells formed an average of 91.5±29.7 per 10^4^ cells per CFU. On the other hand, CD146 negative portion did not show any colonies (*P*<0.001). Both resources showed cells with flowcytometric antigen panel and differentiation potentials conforming to MSC definition.

**Conclusion:**

The entire CFU of PPSCs were formed within CD146 enriched portion. It seems that CD146 enrichment may reduce the number of possible fibroblasts of the pulp polyps and may further homogenize the culture of the PPSCs.

## Introduction

Stem cells are the main sources of cells needed for tissue regeneration. Virtually, they are isolated from any tissue, such as the skin, brain, myocardium, bone marrow and dental pulp [1]. Some have shown promising *in vitro* expansion potentials [2], while others have been proved to have none [3]. Their stemness potentials are affected by many factors including age, disease states, exercise, *etc.* [4]. 

Multipotent mesenchymal stromal cells (MSCs), previously known as mesenchymal stem cells [3], are clonogenic, plastic adherent cells with multiple differentiation capacity into mesenchyme and/or even non-mesenchyme lineages, such as the cardiomyocytes, osteoblasts, hepatocytes and adipocytes [5]. Traditionally, MSCs are isolated from the bone marrow; however, they can be obtained from other tissues such as granulocyte colony-stimulating factor (GCSF)-mobilized peripheral blood [6], adipose tissue [6], umbilical cord blood [7, 8] and dental pulp stem cells (DPSCs) [9]. Human dental pulp and its surrounding tissues are derived from the ectomesenchyme, a tissue resulting from interaction of the neural crest cells and mesenchymal tissues in the embryonic period. In recent years, dental ectomesenchymal tissues such as the periodontal ligament stem cells have been extensively searched for stem cells [10], but only few studies have investigated the pathologically affected tissues for the presence of stem cells. 

**Figure1:**
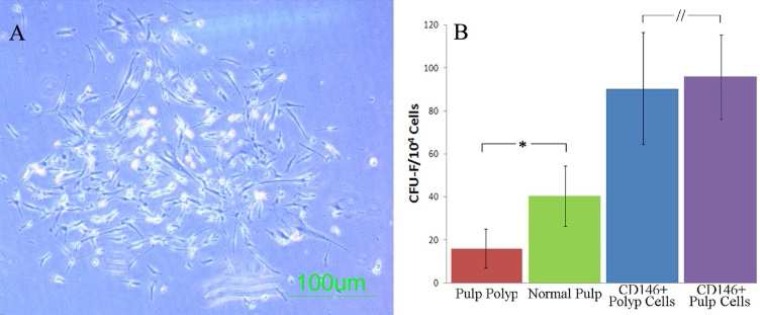
Pulp polyp derived cells: *A)* A single cell derived colony from pulp polyp derived cells formed within CFU-Fibroblast assay; *B)* Comparison of clonogenic efficiency of CD146+ sorted and unsorted pulp polyp and dental pulp derived cells show that unsorted normal pulp has higher potentials which disappears after CD146 sorting. (* difference significant, // difference not significant

Recently, the presence of STRO-1 (a cell surface antigen expressed by stromal elements in human bone marrow) positive cells in chronic hyperplastic pulpitis tissue sections was shown [11] and it was demonstrated that isolation of these cells can lead to cell cultures with stem cell properties similar to that of DPSCs [12]. Unfortunately, the cell isolates of these tissues show mixed and heterogeneous cultures with low colony forming potentials. 

CD146 was one of the first markers explained for DPSCs. Primarily, it was demonstrated to be a marker for pericytes, but it was shown later to be present on DPSCs. Interestingly, it was demonstrated that the entire colony forming units of DPSCs cultures are limited to the CD146 positive portions. As a result, CD146 enrichment can potentially homogenize the cultures of DPSCs and enhance the colony forming potentials of their cultures [13]. Here, we have designed a study to see whether CD146 enrichment can help purify the stem cells from heterogeneous cultures from the pulp polyp derived stem cells (PPSCs).

## Materials and Methods

***Preparation of single cell suspension from the pulp polyps and normal pulps***

All the patients gave their informed written consent. The protocol was approved by the ethics committee of Shiraz University of Medical Sciences and the study conformed to the deceleration of Helsinki for working with human subjects. The pulp polyps were collected from 4 permanent molars of the patients (16-28 years of age). The pulp polyp tissues were removed and digested as explained in other studies [12]. Briefly, the tissues were sliced and underwent enzymatic digestion with a 3 mg/mL solution of collagenase type I (Sigma, St. Louis, MO, USA) and 4 mg/mL of dispase type II (Sigma, St. Louis, MO, USA) for 1 h with occasional shaking [12]. 

Human third molars were collected from the young adults aging 20-25 years. DPSCs were isolated according to previously-published methods, with some modification [9]. 

The obtained single cell suspension from both resources underwent magnetic cell sorting for CD146 and then the negative and positive portions underwent flowcytometric analysis for evaluation of the magnitude of purification, or were suspended in the media to assess colony forming potential or cultured for further analysis.

***Magnetic cell sorting of CD146+ cells***

Magnetic activated cell sorting (MACS) was designed in order to purify the stem cells from the fibroblasts with separation of CD146 stem cells [13]. Primary cells isolated from tissue digestions, from both healthy pulps and pulp polyps, underwent dead cell elimination using dead cell removal kit (Miltenyi Biotec, Bergisch Gladbach, Germany) to decrease the chance of non-specific cell bindings of the antibodies. Then the cells (2.5±0.7×10^5^ cells) were labeled with fluorescein isothiocyanate (FITC)-conjugated CD146 antibody (Becton Dickinson, San Jose, CA, USA) followed by labeling the target cells with anti-FITC microbeads regarding the manufacturer’s instructions. The cell suspension was loaded onto a mean and covariance structures (MACS) analyses of cross-cultural data: MACS column separator. The magnetically labeled CD146+ cells were retained on the column while the unlabeled cells ran through. The isolated cells were used for the next steps. 

**Figure 2:**
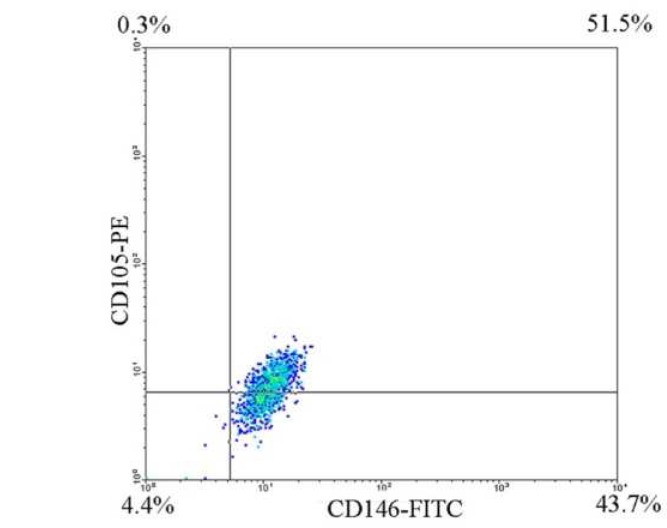
Flowcytometric analysis showed that CD146 cell sorting was done with high purity. The CD146+ portion showed to be partially positive for CD105

***Colony forming assay***

To assess the single cell derived colony formation efficiency, both CD146 sorted and unsorted cells from healthy pulps or pulp polyps were seeded into 6-well plates at a live cell concentration of 1000 cells/mL within -MEM (Gibco/Invitrogen, Carlsbad, CA, USA) supplemented with 4 mM GlutaMAX (Gibco/Invitrogen, Carlsbad, CA, USA), 100 U/mL penicillin, 100 µg/mL streptomycin (Gibco/Invitrogen, Carlsbad, CA, USA), and 20% FBS (Gibco). The cells were cultured at 37^º^C in 5% CO_2_ and 90% humidity. Single cell derived colonies were defined as those units with more than 50 cells. The number of colonies was counted on the day before the colonies were merged, or as late as 14 days after the culture.

***Culturing the isolated cells***

The single cell suspensions from both CD146 sorted and unsorted cells from healthy pulps or pulp polyps were plated in uncoated culture flasks (Orange Scientific) within the above-mentioned media and condition. Unattached cells and debris were then removed and fresh medium added to the adherent cells. Culture media was changed twice weekly until the flask reached 80% of confluency. Then the cells were released with 0.25% trypsin-EDTA solution (Gibco/Invitrogen, Carlsbad, CA, USA) and sub-cultured. These cells were passaged three times before undergoing differentiation or flowcytometric analysis. 

***Flowcytometry***

To analyze the purity of the magnetic cell sorting, the cells were incubated for 30 min in a dark environment with the following anti-human antibodies: CD146-FITC (Becton Dickinson, San Jose, CA, USA), and CD105-PE (Serotec, Kidlington, England). 

To analyze the cell surface antigen expression of the cultured cells, we used the following anti-human antibodies: CD90–Allophycocyanin (APC), CD34-FITC, CD56-Phycoerythrin (PE) and CD45-Peridinin chlorophyll protein (PerCP) (Miltenyi Biotec, Bergisch-Gladbach, Germany), CD14-FITC, CD166-PE, CD44-FITC, CD146-FITC, HLA-DR-PerCP, CD73-PE (Becton Dickinson, San Jose, CA, USA), CD105-PE (AbD Serotec, Kidlington, Oxford, UK) and STRO1-pure (With secondary anti-IgM FITC conjugated IgG, Santa Cruz Biotechnology Inc., Santa Cruz, CA, USA), as previously described [12]. 

Flowcytometric analysis was performed on a FACS Calibur instrument (BD Biosciences, San Jose, CA, USA), using the cell quest as data acquisition software. The Win MDI 2.8 software (Purdue University, West Lafayette, IN, USA) was used for the data analyses.

***Differentiation assays***

To evaluate the differentiation potentials of the cultured cells, osteogenic and adipogenic media were used, as previously described [9, 12]. Briefly, for osteogenic differentiation, the cells were cultured in NH Osteo Diff Medium (Miltenyi Biotec GmbH, Bergisch Gladbach, Germany) for 3 weeks according to the manufacturer’s guidelines and medium exchange was performed twice a week. To approve the differentiation, after appropriate morphological changes, the cells were stained with Alizarin red (Sigma, St. Louis, MO, USA). 

For adipogenic differentiation, the cells were cultured in Mesen Cult medium (Stem Cell Technologies Inc, Vancouver, BC, Canada) supplemented with 10% Adipogenic Stimulatory Supplements (Stem Cell Technologies Inc, Vancouver, BC, Canada) for 3-4 weeks and half of the medium was exchanged only when the color of the media changed to yellow. To approve the differentiation, after appropriate morphological changes, the cells were stained with Oil red (Sigma, St. Louis, MO, USA). 

The significance of the differences between the results among groups was assessed using independent t-tests. A two-sided *P*-value<0.05 was considered statistically significant. All statistical analyses were performed using the statistical Package for Social Sciences version 17.0 (SPSS Inc., Chicago, IL, USA).

## Results

***Colonogenic efficacy***

To assess single the cell derived colony formation (CFU-F assay), only colonies with more than 50 cells were considered in colony enumeration (Figure 1A). Normal pulp tissue derived cells formed higher colonies (42.5±16.8 per 10^4^ cells) than the pulp polyp (17.75±8.9 per10^4 ^cells) (*P*=0.015). The CD146 positive portion of the polyp derived cells formed an average of 91.5±29.7 CFU-Fs. Each 10000 CD146 positive normal pulp cells could form 97.25±17.7 CFUs. CD146 portion of both PPSCs and normal DPSCs had the same colony forming potential with no significant difference (*P*=0.56) (Figure 1B). On the other hand, CD146 negative portion of both resources did not show any colonies. Colony forming efficacy of CD146 positive cells was significantly higher than CD146 negative ones (*P*<0.001).

**Figure 3.**
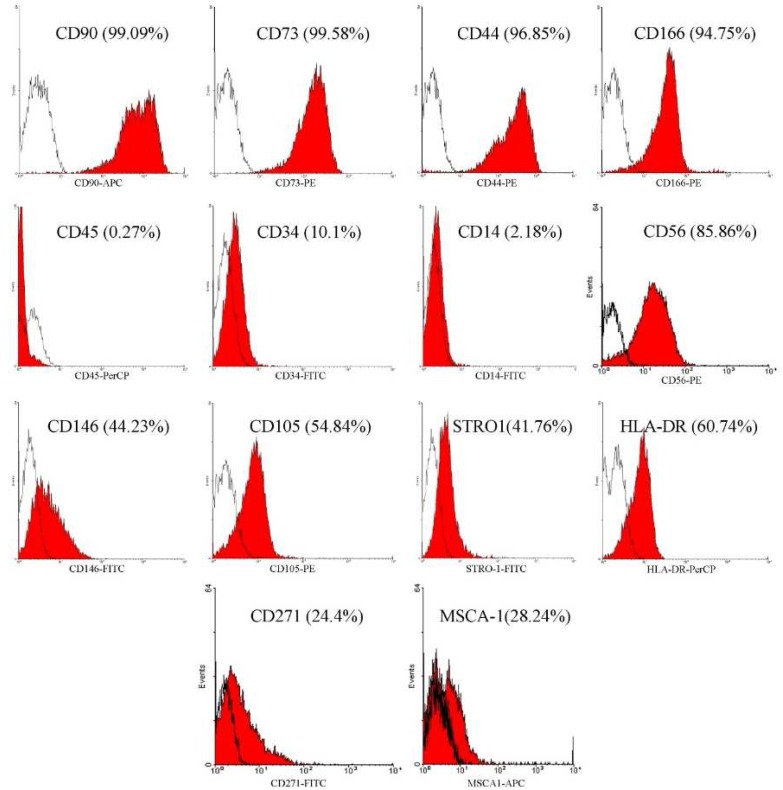
Flow cytometric analysis of PPSCs in vitro revealed a high level expression of CD44, CD166, CD90, and CD73 accompanied by low frequency for surface antigens: CD14, CD34 and CD45. But these cells show a heterogeneous phenotype for CD146, CD105, HLA-DR and STRO-1

***Culture characteristics***

Two days after the initial seeding, both PPSCs and normal DPSCs were attached to the plates with appropriate fusiform-like appearance (Figure 1A). These cells became confluent within 14-20 days with a typical fusiform fibroblast-like appearance.

***Flowcytometric results***

The purity of CD146 positive sorted cells was shown to be 90.96±3.27% for the normal dental pulp cells and 93.08±1.12%for the pulp polyp cells (Figure 2).

Flowcytometric analysis of the cells harvested from the pulp polyp derived cultures showed that the cells were positive for mesenchymal markers such as CD44, CD166, CD90 and CD73, and they were negative for surface molecules CD14, CD34, and CD45. These cells show a heterogeneous phenotype for CD146, CD105, HLA-DR, STRO-1, CD271, MSCA1 and CD56 (Figure 3). 

These results are comparable to the normal DPSCs which were strongly positive for CD44, CD73, CD90, CD105, CD146 and CD166. DPSCs were negative for CD34, CD45, CD14 and HLA-DR, also showing heterogeneous phenotype for STRO-1, CD271, MSCA1 and CD56.

***Differentiation assay***

**Figure 4:**
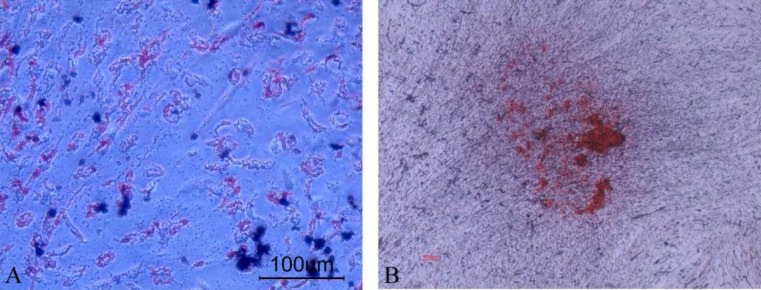
Differentiation of pulp polyp derived cells. A) Lipid vacuoles stained with oil red o stain after adipogenic differentiation; B) Osteogenic differentiation with calcium deposition identified by alizarin red staining

Adipogenic differentiation was done by both morphological and staining criteria. One week after seeding in the adipogenic medium, the cells showed small isolated vacuoles that increased in number and size with time and all of them were stained by Oil red (Figure 4A).

The earliest evidence of MSC differentiation to osteoblasts was matrix depositions around the cells in second. Full differentiation to osteoblasts lasted for 4 weeks. Mineralization was documented by alizarin red staining (Figure 4B). 

## Discussion

In the present study, it was revealed that the entire colony forming units of the pulp polyp derived cells were within CD146 positive portion. This shows that the stem cell portion of the PPSCs is present only within this population. Currently, the main source of cells for dental tissue regeneration is DPSCs. However, their access needs extraction of the teeth with with a healthy and normal pulp. Consequently, they are not viable options for all the times. So, extraction of the stem cells from pathologically affected teeth would often be another option. Previously, Wang and *et al.* [14] showed that the pulps affected by irreversible pulpitis contained putative stem cells. The same is depicted in inflamed periapical tissue [15] and inflamed periodontal ligament [16]. We have shown for the first time that totally pathologically formed dental tissues such as the hyperplastic pulpitis (pulp polyps) also contain cells with stemness properties [12]. The cells isolated from the pulp polyp could successfully form fibroblast CFU with appropriate cell surface antigen marker panel and differentiation potential, all fulfilling the criteria from international society of cell therapy for MSC definition [17]. However, their potential for CFU is lower than the healthy pulp and their cultures show a heterogeneous phenotype. 

Routine cultures of DPSCs which are simply achieved by direct culturing of the normal pulp tissue derived single cell suspensions are proved to be very heterogeneous [18]. This may be explained by the presence of mature cells including the fibroblasts [19]. Currently, we know that many of the previously explained parameters such as plastic adherence, spindle-like morphology or cell surface markers such as CD14, CD34, CD44, CD45, CD73 and CD105 that were used for defining mesenchymal tissues derived stem cells cannot differentiate them from fibroblasts [20]. Recently, some discriminating factors have been purposed. Alt *et al.* [20] showed that CFU capacity may serve this purpose**. **In another study by Halfon *et al.* [21], CD146 expression was shown to be restricted to the stem cell portion of the adipose tissue derived cultures and absent on the fibroblasts. Furthermore, it was indicated by Zannettino *et al.* [22] that the entire CFUs are limited to the CD146 positive cells. These findings may prove that CDl46 enrichment can help one to discriminate the true mesenchymal stem cells from the fibroblasts. Accordingly, we showed in a prospective study for the first time that CD146 isolation approach can purify both stem cells and fibroblasts from a dental pulp tissue as well [13]. Therefore, CD146 enrichment may serve as a tool to purify the stem cells from the pulp polyps as well. It should be noticed that if a marker is useful for separation of the stem cells from one source, it may not be as efficient for other sources. For example, CD271 is an excellent marker for isolation of MSCs from the bone marrow but it is not useful for isolation of the same cells from the umbilical cord blood [7] or the GCS-F mobilized peripheral blood [6]. So there was a need to conduct a study to answer the mentioned question. In our study, it was revealed that the entire colony forming units of the polyp cells were within CD146 enriched portion. It seems that CD146 enrichment may help reducing the number of possible fibroblasts seen in the histological sections of the pulp polyps and may further homogenize the culture of the cells from this heterogeneous pathologically formed tissue. 

Using PPSCs for clinical implications faces some limitations. As pulp polyps are exposed to oral cavity contents, the possibility of contaminations becomes higher. For this reason, we used anti-bacterial and anti-fungal supplementations since the moment of tissue collection to the final steps of sample preparation. As another disadvantage, it should be mentioned that there is a higher variability in the number of stem cells within a polyp than a normal pulp as the formation of the polyps follows a pathological process. However, using CD146 enrichment may help reducing these variability.

## Conclusion

According to the results of this comparative study, pulp polyp has appropriate amounts of stem cells and CD146 isolation can help further homogenize their cultures and enhance their stem cell yield. This may help more in using the pulp polyp derived stem cells as a possible source for autologous stem cell therapies.
